# Clinical Analysis of Interventional Therapy for Eight Cases of Extracranial ICA Aneurysm

**DOI:** 10.12669/pjms.37.4.3957

**Published:** 2021

**Authors:** Jian-Feng Xu, Zao-Bin Liu, Tong Wang, Yang Liu

**Affiliations:** 1 Jian-Feng Xu, Neurosurgery Department, The Third Hospital of Mianyang/Sichuan Mental Health Center, Mianyang, Sichuan, 621000, P.R. China; 2 Zao-Bin Liu, Neurosurgery Department, The Third Hospital of Mianyang/Sichuan Mental Health Center, Mianyang, Sichuan, 621000, P.R. China; 3 Tong Wang, Neurosurgery Department, The Third Hospital of Mianyang/Sichuan Mental Health Center, Mianyang, Sichuan, 621000, P.R. China; 4 Yang Liu Neurosurgery Department, The Third Hospital of Mianyang/Sichuan Mental Health Center, Mianyang, Sichuan, 621000, P.R. China

**Keywords:** Aneurysm, Stent, Extracranial ICA, Interventional therapy

## Abstract

**Objective:**

To explore the interventional therapy and clinical efficacy of extracranial ICA aneurysm.

**Methods:**

The clinical data of eight patients with extracranial ICA aneurysm treated by interventional stent implantation from December 2014 to February 2018 in the Neurosurgery Department of the Third Hospital of Mianyang were analyzed. And this research was a retrospective analysis. All patients underwent digital subtraction angiography (DSA) and were diagnosed with extracranial carotid artery aneurysm. These patients, therefore, were treated with interventional stent implantation.

**Results:**

Interventional treatment was successfully conducted on all eight patients. In eight patients, the aneurysm cavity was not developed immediately after angiography, and in one case, the aneurysm cavity was developed with coil-assisted embolization. All the internal carotid arteries were well developed, with no complications such as intraoperative rupture, bleeding and thrombosis occur. Follow-up for three months to two years showed that the patients recovered well, the GOS score was 4 points for patients with cerebral infarction, and the rest reached five points. Follow-up CTA showed no signs of aneurysm recurrence or ICA restenosis.

**Conclusion:**

Interventional stent placement is a preferable and relatively safe method for the treatment of extracranial carotid artery aneurysm with less trauma and short operation time.

## INTRODUCTION

Aneurysms of the extracranial internal carotid aneurysm (EICA) account for an estimated 0.1% to 2% of all carotid procedures and less than 1% of all arterial aneurysms.^1^ The most common underlying causes related to these true aneurysms include atherosclerosis, trauma, fibromuscular dysplasia, infection, connective tissue disorders, and large vessel vasculitis.^2^ EICA generally has no obvious clinical symptoms. Some patients usually show pain, throbbing masses or cranial nerve palsy.^3^ In some medical centers, EICA accounts for only 0.1%-2% of carotid artery related operations.^4^ EICA contributes to high-level factors such as local compression and the formation of mural thrombus shedding, leading to a high incidence of stroke events. Therefore, patients should actively undergo surgical intervention once they are diagnosed with EICA.^5^ For such disease, surgical treatment and interventional therapy have been reported in the past. Endovascular interventional therapy, however, has the characteristics of minimally invasive, less neurovascular injury and fewer postoperative complications, which is often used as the preferred treatment strategy. In this paper, the clinical data of patients with EICA treated by interventional stent implantation in our center were analyzed retrospectively, and the interventional treatment and clinical efficacy were explored.

## METHODS

### Ethical approval

The study was approved by the Institutional Ethics Committee of The Third Hospital of Mianyang/Sichuan Mental Health Center at August 29, 2020, and written informed consent was obtained from all participants.

### Methods

This research was a retrospective analysis. And PASS software was used to calculate the sample size. Of eight patients with EICA treated by interventional stent implantation from December 2014 to February 2018 in the Third Hospital of Mianyang were analyzed retrospectively. Six male and two female patients were enrolled into this group of cases. The age ranged from 41 - 66 years, with an average age of 50.5 years. Among the eight patients, five cases showed dizziness, one case was found to have intracranial aneurysm during follow-up, one case had sudden limb paralysis during CT examination, and one case had brain stem hemorrhage in rehabilitation period.

### Imaging examination

All patients were first diagnosed with EICA by CTA, and one patient with cerebral infarction was complicated with contralateral internal carotid stenosis. All patients underwent digital subtraction angiography (DSA) and were diagnosed with EICA.

### Therapeutic method

All patients received interventional therapy. 100 mg of aspirin and 75 mg of clopidogrel were taken orally before the operation for 3-5 days. During the treatment, three cases were given local anesthesia, four cases were treated with tracheal intubation general anesthesia, the right femoral artery was punctured and the sheath was inserted, the 8F guide catheter was inserted, the cerebral angiography evaluation was performed first, and the 3D angiography evaluation was performed. Relevant data were measured based on the angiography results, including the diameter from the proximal end to the distal end of the inter carotid artery (ICA) around the aneurysm’s neck, the width of the aneurysm’s neck and the distance from the initial part of the ICA and the skull base. During the operation, heparinization was performed. In the road map mode, the guide wire of five patients passed through the distal end of the ICA at the aneurysm site, and then the stent was delivered and released around the aneurysm neck. Angiography showed that the aneurysm was not developed and the parent artery was unobstructed. In three patients, microcatheter was inserted into the aneurysms under the guidance of micro-guide wire. The coils were embolized, and then the aneurysmal neck was covered with stents. Low molecular weight heparin (LMWH) was injected subcutaneously for three days after the operation. Aspirin was routinely taken orally for six months and clopidogrel for three months. One patient was recommended to take aspirin for a long time due to multiple high-risk factors for cardiovascular and cerebrovascular diseases.

## RESULTS

### Basic information of cases (As shown in Table-I):

**Table-I:** Statistical graph of basic treatment information of patients.

Patient No.	Gender	Age	Proximal diameter of ICA of aneurysm	Distal diameter of ICA of aneurysm	Width of aneurysm neck	Treatment	Brand of stent	Type of stent	Specifications	Complication	Follow-up time	Follow-up results and Rank classification
1	Male	50	4.8	3.9	5	Stent implantation alone	Abbott	Acculink	6mm*8mm*40mm conical stent	None	4 years	Good, 5
2	Female	45	4.9	3.2	4.7	Stent implantation alone	Bard	FVL06040	6mm*40mm	None	2 years	Good, 5
3	Male	66	3.9	3.5	4.9	Stent implantation alone	Bard	FVL06040	6mm*40mm	None	3 years	Good, 5
4	Male	46	5.1	4.05	3.4	Stent placement + coil embolization	EV3	Protege carotid artery stent	1) 6mm*8mm*40mm conical stent 2) Coils : Auxium 6mm *30mm*1	None	2 years	Good, 4
5	Male	41	5.09	3.78	8.67	Stent placement + coil embolization	EV3	Solitare AB	1) Stent : 6mm*30mm 2) Coils : Auxium 7mm*30mm*1,6mm*20mm*1	None	1 years	Good, 5
6	Male	54	4.2	5.3	14.1	Stent implantation alone	EV3	Protege carotid artery stent	7mm*40mm	None	2 years	Good, 5
7	Female	60	5.1	4.5	5	Stent placement + coil embolization	EV3	Solitare AB	6mm*30mm	None	1 years	Good, 5
8	Male	41	5	4.85	5.6	Stent placement + coil embolization	1) EV3 2) BALT	Solitaire AB	1) Stent:6mm*30mm. 2) Coils:Auxium 5mm*15mm*1,3mm*8mm*2;	None	2 years	Good, 5

### Case 1

**Fig.1:**
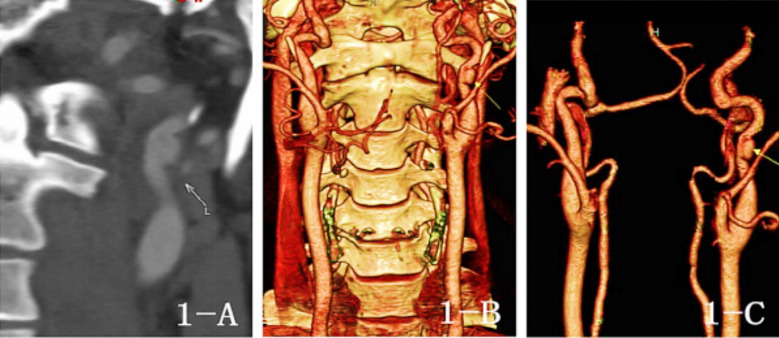
Preoperative CTA images Fig.1-A: (MIP), Fig.1- B, 1-C: (Reconstruction Image) revealed left extracranial carotid artery aneurysm, Size: 5*8mm

**Fig.2:**
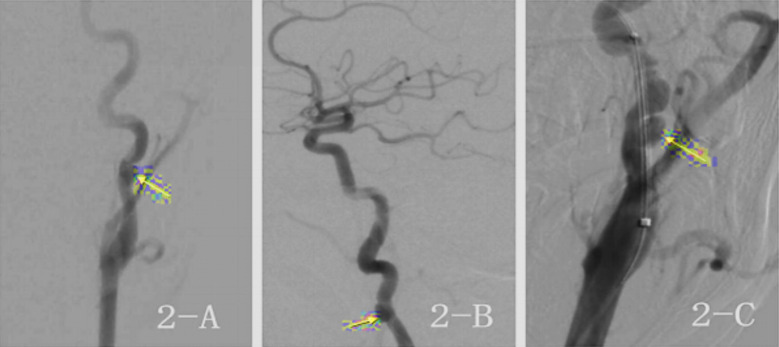
Intraoperative DSA Images Fig.2-A: Left internal carotid angiography (Anteroposterior position), Fig. 2-B: Left internal carotid angiography (Lateral position), Fig. 2-C: Stent Implantation with Parent Artery of Left extracranial carotid artery aneurysm.

**Fig.3:**
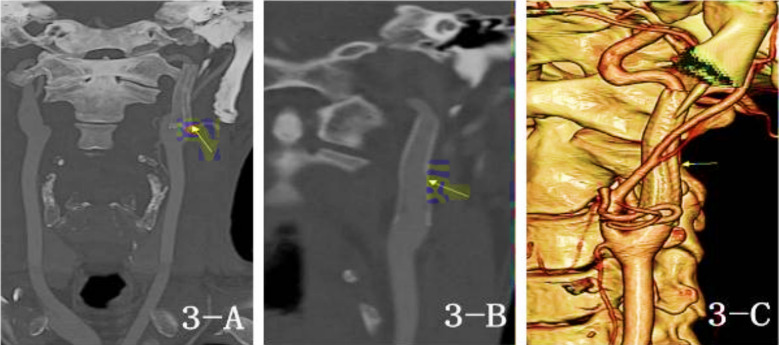
CTA image for 1-year postoperative follow-up Fig.3-A, Fig.3-B: (MIP) and Fig.3-C: (Reconstruction Image) showed the stent had no displacement and the blood flow was unobstructed)

Basic information: male, 50 years old. Chief complaint: pain in the right occiput posterior position and the top of the head for 6+ days. Imaging examination: CTA: left ICA dissecting aneurysm with artery stenosis. DSA: Left extracranial carotid artery aneurysm, about 5*8mm in size, with irregular shape and local carotid artery stenosis (see Fig. 1, 2, 3 and Table-I).

### Case 2

Basic information: female, 46 years old. Chief complaint: Three months after the intracranial aneurysm operation, the right extracranial carotid artery aneurysm was found for 10+ days. Imaging examination: CTA: postoperative performance of right extracranial carotid artery aneurysm, and intracranial aneurysm clipping. DSA: right extracranial carotid artery aneurysm, about 8*4mm in size and irregular in shape.

### Case 3

Male, 66 years old. Chief complaint: paroxysmal amaurosis in left eye for 1 week. Imaging examination: head and neck CTA: dissecting aneurysm of the left ICA Cranial DSA: dissecting aneurysm of the left extracranial ICA.

### Case 4

Male, 46 years old. Chief complaint: dizziness for 9 hours, aggravated with weakness of right limb for 6+ hours. Imaging examination: head and neck CTA: 1. Tumor-like nodules are prominent in the distal segment of the right ICA, suggesting aneurysms. 2. Local lumen stenosis in the cervical segment of the left ICA; 3. A large area of slightly low-density shadow was seen in the left basal ganglia and left parietal lobe. DSA: 1. Aneurysm at the distal end of the extracranial segment of the right ICA, about 6 * 7 mm in size; 2. The stenosis in the middle section of the left ICA has changed.

### Case 5

Male, 41 years old. Chief complaint: sudden weakness of the right limb with slurred speech for 7+ months. Imaging examination: CTA: the lumen of the left ICA at the end of the petrous segment was widened and swelled to the medial navicular shape, showing double lumen, suggesting localized dissection with aneurysm formation. DSA: right extracranial carotid artery aneurysm, about 19*8mm in size and irregular in shape.

### Case 6

Basic information: male, 54 years old. Chief complaint: hypomnesis with fatigue of right upper limb for three months. Imaging examination: CTA: double lumen shadow was seen in the proximal segment of the left ICA, and low-density intimal film was seen in it, suggesting a localized dissecting aneurysm with a range of about 1.67cm. DSA: The dissection of the initial segment of the left ICA has changed, and irregular in shape.

### Case 7

Female, 61 years old. Chief complaint: recurrent dizziness with visual rotation for 20 years, followed by limb shaking for 1 day. Imaging examination: DSA: 1. Left main aneurysm of ICA, about 13*10mm in size.

### Case 8

Male, 42 years old. Chief complaint: headache for 4+ days. Imaging examination: DSA suggests: 1. Localized enlargement of the petrous bone segment of the right ICA; 2. On the right vertebral artery angiography, the irregular enlargement of the distal segment of the vertebral artery was observed.

### Outcomes

Eight patients were successfully treated. One patient complicated with contralateral ICA stenosis was treated with ICA stent implantation. After the operation, LMWH was injected subcutaneously for three days, aspirin was taken for six months and clopidogrel for three months. One patient complicated with multiple high-risk factors of cardiovascular and cerebrovascular diseases suggested taking aspirin for life. In seven patients, the aneurysm cavity was not developed immediately after angiography, and in one case, the aneurysm cavity was developed with coil-assisted embolization. All the internal carotid arteries were well developed, with no complications such as intraoperative rupture, bleeding and thrombosis occur. Follow-up for three months to two years showed that the patients recovered well, the GOS score was four points for patients with cerebral infarction, and the rest reached five points. Follow-up CTA showed no signs of aneurysm recurrence or ICA restenosis.

### Typical case images presentation

Treatment of extracranial aneurysm of left ICA with stent implantation (Case 1), aneurysm marked by arrow.

## DISCUSSION

Extracranial carotid artery includes the common carotid artery, the external carotid artery and the internal carotid artery (ICA) till the skull base.^6^ The EICA is defined as a dilation of 150% or more of the diameter of the expected normal carotid artery.^7^ EICA is rare and accounts for less than 1% of all peripheral artery aneurysms, and the pathogenesis is not yet clear.^8^ It may be related to dissections, atherosclerosis, infection, fibromuscular dysplasia, connective tissue disease and traumatic or spontaneous dissection.^9^,^10^ EICA is mainly located in the bifurcation of carotid artery or the distal end of ICA^11^, accounting for 0.4%-4% of peripheral aneurysms and 1.5% of all carotid artery revascularization. The incidence of EICA in male is higher than that in female, with the ratio of about 3:1, and the average age of 61.9 years.

The development of EICA is gradually accompanied by neurological dysfunction, such as transient ischemic attack, aneurysm rupture and bleeding, thrombosis, cerebral infarction, peripheral nerve compression and so on. Studies by Jin ChunXiang et al. show that^12^ even if EICA has no relevant clinical manifestations, imaging examination suggests that early surgical treatment should be performed if cerebral blood supply is affected. Therefore, once the diagnosis is made, active surgical intervention is necessary. EICA is divided into conservative treatment and surgical intervention. Conservative treatment mainly includes anticoagulation and antiplatelet aggregation. It has been reported that, however, anticoagulation is ineffective, because the incidence of stroke is still 50% even under anticoagulation. Therefore, surgical treatment should still be the preferred treatment even for asymptomatic patients.

Surgical treatment mainly includes: surgical treatment, endovascular interventional therapy, surgical treatment combined with endovascular therapy, etc. Surgical treatment of EICA has been conducted for many years. Welleweerd JC et al. believe that^13^ surgery treatment is the accepted treatment method for symptomatic EICA, and complete aneurysm resection combined with vascular reconstruction was once considered as the gold standard method. However, surgical treatment has the disadvantages such as large trauma, easy to produce neurological deficits after surgery, etc.^14^ Surgical treatment combined with endovascular therapy is mainly aimed at EICA with large and near skull base and severe distortion of ICA.

Endovascular interventional therapy has the characteristics of minimally invasive, less neurovascular injury and fewer postoperative complications.^15^ Therefore, endovascular interventional therapy is more widely used in the treatment of EICA. Wellewerd Janna C et al.^16^ performed endovascular therapy on seven patients with symptomatic EICA. After the operation, the patients had no local or central nervous symptoms, and the thrombosis in the aneurysm was completely occluded, which proved that endovascular therapy of EICA aneurysms was technically feasible and safe. In our study, the internal carotid artery was well developed in all patients without intraoperative rupture, bleeding, thrombosis and other complications, which further verified the safety of endovascular interventional therapy. Han Daniel K et al.^17^ reported that a patient with multiple cranial EICA was successfully treated with stent-assisted coil embolization. Haruma J et al.^18^ performed endovascular treatment for a patient with EICA who had undergone endovascular resection and had a recurrence. After the operation, the parent artery was unobstructed and the aneurysm was not developed. The follow-up examination three months after the operation showed that EICA was completely occluded. Therefore, endovascular treatment can also be applied to patients undergoing re-operations to reduce the risk of surgery caused by local anatomical abnormalities. In recent years, endovascular interventional therapy has been more widely used in the treatment of EICA.

In the interventional therapy of EICA, stents, coils and balloons are commonly used. Stents include balloon expandable stents, self-expanding stents, and covered stents. In the treatment of this part of aneurysm, two cases were treated with covered stents, six cases with self-expanding stents, including three cases with carotid artery stents. Four patients were treated with stent isolation alone, and four patients were treated with coil-assisted stents for EICA. In recent years, the flow-diverting stent has been gradually applied to the treatment of EICA and achieved good results.^19^ According to Robijn SMM et al.^20^, a 21-year-old male patient with EICA was treated with flow-diverting stent. Six months after the operation, DAS showed that the aneurysm was occluded well without complications. In the treatment of EICA, coil embolization alone is rarely used, but usually combined with stent for endovascular treatment. Our research has further enriched the clinical data and research references for the application of intravascular interventional techniques in extracranial carotid aneurysms. It is center recommended by our center that for patients with extracranial ICA whose imaging changes are mainly in the form of dissection, carotid artery stents, covered stents or flow-diverting stents are the first choice to isolate the dissection and repair the blood vessels. Those with tumor cysts can be treated with stents (such as Solitaire stents) plus coil embolization. The sample size needs to be further expanded, and prospective control studies need to be conducted in the future owing to the small sample size and lack of long-term follow-up in this study.

### Limitations of the study

There is no unified standard for diagnosis and treatment of EICAA, which needs to be further improved.

## CONCLUSION

Interventional stent implantation or coil-assisted embolization is a preferable and relatively safe method for the treatment of EIAA with less trauma and short operation time. It can be used to assess the blood flow and has a good follow-up clinical efficacy.

### Authors’ Contributions:

**JFX and ZBL** designed this study and prepared this manuscript, and are responsible and accountable for the accuracy or integrity of the work.

**YL** collected and analyzed clinical data.

**TW** significantly revised this manuscript.

**JFX** **and** **ZBL** both considered as first author.
